# Posttraumatic Growth After Struggling With the Loss of a Parent in Young Adulthood

**DOI:** 10.1177/00302228231187175

**Published:** 2023-06-29

**Authors:** Tina Lundberg, Kristofer Årestedt, Mariann Olsson, Anette Alvariza, Ulla Forinder

**Affiliations:** 1 Department of Health Care Sciences/Palliative Research Centre, 7643 Marie Cederschiöld University, Stockholm, Sweden; 2 Function Area in Social Work and Health, Karolinska University Hospital, Stockholm, Sweden; 3 Faculty of Health and Life Sciences, 427813 Linnaeus University, Kalmar, Sweden; 4 Department of Research, Region Kalmar County, Kalmar, Sweden; 5 Department of Neurobiology, Care Sciences and Society/Division of Family Medicine and Primary Care, 27106 Karolinska Institutet Huddinge, Stockholm, Sweden; 6 Department of Research and Development/ Palliative Care, Stockholms Sjukhem, Stockholm, Sweden; 7 Department of Social Work and Psychology, 3485 Gävle University, Gävle, Sweden

**Keywords:** bereavement, palliative care, parental death, posttraumatic growth, young adult

## Abstract

This study aims to examine posttraumatic growth and its associations with parental bereavement among adolescents and young adults. Fifty-five young adults who had lost a parent to cancer at least 2 months earlier and were about to attend a support group at a palliative care service were recruited. Data was collected through questionnaires before support group participation, about 5–8 months after the loss and at a 6-month follow-up, about 14–18 months after the loss. The result shows that the young adults experienced posttraumatic growth, mostly in the domains Personal strength and Appreciation of life. Posttraumatic growth was associated with bereavement outcomes, especially life satisfaction, a feeling of meaning in future life and psychological health. The result is of value for health care professionals as it adds information about the importance of supporting constructive rumination to enhance the possibility to positive psychological change after a parent’s death.

## Introduction

The loss of a parent is a challenging experience and can be particularly traumatic in adolescence and young adulthood, a period in life which is already characterized by comprehensive life changes (Arnett et al., 2014). Though research has shown several psychosocially troublesome consequences of parental loss among adolescents and young adults, such as psychological distress (Farella Guzzo & Gobbi, 2021; Lundberg et al., 2018a, 2018b, 2020, 2022), the traumatic experience might also bring about positive psychological changes in life, known as posttraumatic growth (Calhoun et al., 2010; Gerrish et al., 2009).

Posttraumatic growth may occur as a result of the struggles to handle the difficulties after a traumatic event, e.g., death of a parent. The concept posttraumatic growth is depicting an enhanced feeling of inner strength, closer relationships with family or friends, and a greater appreciation of life (Tedeschi & Calhoun, 2004). The theoretical foundation is to be found in Janoff-Bulman’s theory of shattered assumptions (Janoff-Bulman, 1992). As a trauma shatters a person’s assumptive world, there might be need for a new lifeworld, a renewal which has the potential of leading to a better functioning than before. Thus, posttraumatic growth and posttraumatic stress syndrome or other trauma-related consequences could be experienced at the same time (Calhoun & Tedeschi, 2006). In fact, some degree of lasting distress is a prerequisite for posttraumatic growth (Tedeschi & Calhoun, 2004).

Previous research on adults has shown that also for such traumatic events as the loss of a parent, a disruption of the assumptive world and rumination leading to eventual posttraumatic growth, is common while struggling with the loss (Calhoun et al., 2010). Posttraumatic growth is shown to increase with age (Vishnevsky et al., 2010) and more often experienced by women than men (Tedeschi & Calhoun, 1996; Zhou, Yu, et al., 2018). Tedeschi and Calhoun (2004) also show that social support may facilitate posttraumatic growth. Social support may reduce stress and accordingly enable posttraumatic growth after a traumatic event like loss of a loved one (Linley & Joseph, 2004).

Posttraumatic growth among adolescents and young adults is increasingly studied in relation to different types of natural or man-made disasters (McClatchey & Raven, 2017). However, only a few studies address posttraumatic growth among adolescents and young adults in bereavement. In addition, the findings in these studies concerning what might be of importance are contradictory. (Brewer & Sparkes, 2011; Şimşek Arslan et al., 2022; Wolchik et al., 2009). A systematic review involving six studies on bereaved young persons between 6 and 25 years demonstrates that posttraumatic growth is experienced after parental loss, and that factors such as age, cause of death, the closeness of the person who was lost as well as the time since the loss are factors that may impact posttraumatic growth (Şimşek Arslan et al., 2022). However, none of these findings regarding associations are consistent across studies (Michael & Cooper, 2013; Şimşek Arslan et al., 2022; Wolchik et al., 2009). Therefore, there is a need to further examine posttraumatic growth and its associations among parentally bereaved individuals. To fill the knowledge gap, this study aims at examining posttraumatic growth and its associations with bereavement in a sample of adolescents and young adults who lost a parent to cancer.

## Methods

### Design

The present study is based on data from a larger longitudinal study which aimed to investigate psychosocial well-being among young adults who attended a support group after parental loss. Data was collected at three time points from October 2011 through July 2016 (Lundberg et al., 2018a, 2018b, 2020, 2022; Olsson et al., 2017). The present study is based on the first and the last assessment.

### Ethical Considerations

Bereaved young adults are a vulnerable group and the voluntariness and confidentiality of the participants was maintained throughout the research. A screening-procedure was applied to detect participants with mental health problems at follow-up; 10 participants were identified and followed up regarding continued support needs. The study follows the Declaration of Helsinki and was approved by the Regional Ethical Review Board of Stockholm, Sweden (No. 2011/419-31/5).

### Sample and Procedure

Participants were recruited from three palliative care services in Sweden. Adolescents and young adults (onwards referred to as young adults), 16–28 years, who had lost a parent to cancer more than 2 months earlier and were about to attend a support group at the palliative care services were invited to participate. The purpose of the support groups was to offer a place where the participants could share their grief and support each other. The groups were led by two mentored professionals with experience from palliative care. The themes that were covered concerned the death of the parent, the new circumstances of life, their grief and existential thoughts, experiences of support, memories of the parent and thoughts about the future (Henoch et al., 2016; Olsson et al., 2017).

The participants were recruited by the group leaders through oral and written information. A baseline questionnaire was distributed by the group leaders and was completed before the support group participation about 5–8 months after the loss. A second questionnaire was completed about 8–12 months after the loss, directly after the final support group meeting. A third questionnaire was completed about 14–18 months after the loss, 6 months after the support group participation had ended. Reminders was sent twice to those who had not responded. The present study uses data from baseline and the 6-month follow-up. In total 77 young adults were included in the study of which 55 young adults completed the 6-month follow-up and were included in this study.

### Data Collection

The questionnaires included demographic questions (e.g., age and gender) as well as validated instruments and single items to measure posttraumatic growth, bereavement outcomes, bereavement stressors, and sources of social support. The integrative risk factor framework for the prediction of bereavement outcome (Stroebe et al., 2006) was used to guide the composition of the questionnaire. The framework includes the Cognitive Stress Theory (Lazarus, 1984) and the Dual Process Model of Coping with Bereavement (Stroebe & Schut, 1999). The framework offers a way of understanding how individuals differ in their ways of adjusting in bereavement. Variables used in this study to investigate such associations are further described below.

#### Posttraumatic Growth

The Posttraumatic Growth Inventory (PTGI) was used to measure posttraumatic growth (Tedeschi & Calhoun, 1996). The instrument measures positive change that may follow in the aftermath of a negative event. It contains 21 items divided into five domains: Relating to others (seven items), New possibilities (five items), Personal strength (four items), Spiritual change (two items) and Appreciation of life (three items). The inventory is answered on a 6-point Likert scale, ranging from ‘Not at all’ (0) to ‘To a very large extent’ (5). The responses within each domain is summarised and divided with the number of items. Thus, all scales have a possible range between 0 and 5, with a higher score indicating more posttraumatic growth. The PTGI has demonstrated satisfactory measurement properties (Tedeschi & Calhoun, 1996) and is frequently used in research (Morris et al., 2020; Shakespeare-Finch et al., 2013). In the present study, internal consistency measured by Cronbach’s alpha was 0.85 for the dimension Relating to others, 0.79 for New possibilities, 0.75 for Personal strength, 0.57 for Spiritual change, and 0.63 for Appreciation of life.

#### Bereavement Outcomes

*Bereavement outcome* was measured by a single item derived from a Danish evaluation of support groups for young adults (Børn, Unge & Sorg, 2012a; 2012b). The item regarded the statement ‘My life in the future will be meaningful’. The item was answered on a 7-point numeric rating scale, ranging from ‘High agreement’ (1) to ‘Low agreement’ (7). Bereavement outcomes was further examined by the following instruments:

The Life Satisfaction checklist (LiSat-11) contains 11 single items measuring satisfaction with life as a whole, work, economy, leisure, contacts with friends, sexual life, ability to manage self-care, family, partner relation, physical health, and psychological health. The items are answered on a 6-point Likert scale, ranging from ‘Very unsatisfying’ (1) to ‘Very satisfying’ (6). In conformity with the constructors’ instructions, ratings were dichotomized; score 1–4 represent ‘Not satisfied’ and score 5–6 represent ‘Satisfied’. LiSat-11 has previously shown satisfactory measurement properties (Fugl-Meyer et al., 2002). Only two of the items, satisfaction with life as a whole and satisfaction with psychological health, were used in the present study.

The Hospital Anxiety and Depression Scale (HADS) consists of 14 items divided on two subscales that measure symptoms of anxiety and depression respectively (Zigmond & Snaith, 1983). Each item has four response options ranging from 0 to three and the subscale scores has a possible range between 0 and 21; higher scores indicate higher symptom levels. The HADS is widely used and has demonstrated satisfactory measurement properties (Bjelland et al., 2002). In the present study, internal consistency measured by Cronbach’s alpha was satisfactory for both subscales, 0.73 for anxiety and 0.81 for depression.

#### Bereavement Stressors

*Bereavement stressors* were measured by a set of single items developed specifically for the present research project. The items were developed with guidance from the integrative risk factor framework for the prediction of bereavement outcome. According to the framework, the coping process in bereavement is characterized by an oscillation between loss-oriented bereavement stressors (i.e., relating to the loss itself) and restoration-oriented bereavement stressors (i.e., relating to the subsequent adjustment following the loss) (Stroebe et al., 2006). Bereavement stressors related to loss were measured by items such as time of awareness of the parent’s cancer, of awareness of the impending death of the parent as well as the quality of the relation with the deceased parent. Bereavement stressors related to restoration after the loss included items such as the quality of the relationship with the still living parent.

#### Sources of Social Support

*Sources of social support* were measured by a set of single items, derived by using the integrative risk factor framework for the prediction of bereavement outcome (Stroebe et al., 2006). The sources of social support related to with whom the grief was shared, such as the parent before death, the still living parent, a sibling, a partner, friends or no one. Furthermore, the sources of social support concerned if support was received from a professional, the numbers of sources of support (i.e., professionals, family/friends, support group, no one), or if no support was received.

### Statistical Analysis

Descriptive statistics was used to present the characteristics of participants and study variables; interval level data are presented with mean and standard deviation, ordinal data with median and quartiles, and non-ordered categorical data with frequencies.

Spearman’s correlation coefficients were used for examining the associations between posttraumatic growth and bereavement. Since the sample size was small, we have reported correlation coefficients that correspond to the Cohen’s r effect size rather than statistically significant correlations. The Cohen’s r effect size was interpreted as: 0.1–0.3 small, 0.3–0.5 medium, and >0.5 large (Cohen, 1992). All statistical analyses were conducted with SPSS Statistics 26.0 (IBM Corp., Armonk, NY, USA).

## Results

### Participants Characteristics at Baseline

The final sample included 55 young adults, 46 (84%) females and 9 (16%) males. Their mean age was 23 years (range = 16–28), and most of them worked and/or studied (*n* = 51, 93%). More information about the participants is presented in Table 1.

**Table 1.** Participant Characteristics at Baseline (*n* = 55).

	Valid Data, *n*	Total Sample
Age, Mean (SD) [range]	55	23 (3.1) [16–28]
Gender, *n* (%)	55	
Female		46 (84)
Male		9 (16)
Has a partner, *n* (%)	55	
No		27 (49)
Yes		28 (51)
Living conditions, *n* (%)	55	
Living alone		19 (35)
Living with partner		15 (27)
Living with parent		19 (35)
Living with sibling		2 (4)
Working conditions, *n* (%)	55	
Working		24 (44)
Studying		21 (38)
Working and studying		6 (11)
Unemployed		2 (4)
Sick leave		1 (2)
Parental leave		1 (2)
Deceased parent, *n (*%)	55	
Mother		32 (58)
Father		23 (42)

### Posttraumatic Growth

Every young adult reported posttraumatic growth; four in three of the domains of the PTGI, 11 in four of the domains and 40 in all domains. In two domains of posttraumatic growth a few young adults reported no growth, Spiritual change (*n* = 9) and New possibilities (*n* = 3). The domain with the highest level of growth was Appreciation of life (Mdn = 3.3), and the priorities of what is important in life and the appreciation of the value of the own life was reported to had changed most. The domain with the second highest level of growth was Personal strength (Mdn = 3), in which the knowledge that difficulties could be handled and the discovery of being stronger than thought had changed most. The lowest level of growth was reported within Spiritual change (Mdn = 1.5) (Table 2).

**Table 2.** PTGI Domains and Items at Follow-up 2 (*n* = 55).

Mother’s/Father’s Death Have Contributed to	Mdn (q1–q3)
PTGI total	2.3 (1.7–3.1)
Relating to others	2.3 (1.6–3.6)
6. Knowing that I can count on people in times of trouble	2 (2–3)
8. A sense of closeness with others	3 (1–3)
9. A willingness to express my emotions	3 (1–3)
15. Having compassion for others	3 (0–4)
16. Putting effort into my relationships	4 (2–4)
20. I learned a great deal about how wonderful people are	3 (0–4)
21. I accept needing others	3 (1–4)
New possibilities	2.2 (1.2–2.8)
3. I developed new interests	2 (0–3)
7. I established a new path for my life	2 (0–3)
11. I’m able to do better things with my life	3 (2–4)
14. New opportunities are available which wouldn’t have been otherwise	1 (0–3)
17. I’m more likely to try to change things which need changing	3 (2–4)
Personal strength	3 (2–3.8)
4. A feeling of self-reliance	3 (1–4)
10. Knowing I can handle difficulties	4 (2–5)
12. Being able to accept the way things work out	3 (1–4)
19. I discovered that I’m stronger than I thought I was	4 (2–5)
Spiritual change	1.5 (0.5–2.0)
5. A better understanding of spiritual matters	2 (1–4)
18. I have a stronger religious faith	0 (0–1)
Appreciation of life	3.3 (2.0–4.0)
1. My priorities about what is important in life	4 (3–5)
2. An appreciation for the value of my own life	4 (3–5)
13. Appreciating each day	2 (1–4)

### Correlations Between Posttraumatic Growth and Demographic Factors

Younger age was associated with higher levels of growth within all domains of posttraumatic growth except for Relating to others and Appreciation of life. Males reported lower levels of growth in PTGI total and Relating to others, compared to females. However, the effect sizes were small (Table 3).

**Table 3.** Associations Between Posttraumatic Growth and Demographic Factors, Bereavement Stressors and Bereavement Outcomes (*n* = 55).

	PTGI Tot	Relating to Others	New Possibilities	Personal Strength	Spiritual Change	Appreciation of Life
Demographic factors						
Age	−.16		−.14	−.13	−.11	
Male sex (female as reference category)	−.11	−.15				
Bereavement outcomes						
Meaning in future life	.20	.19	.13	*.37*	−.24	*.35*
Satisfaction with life as a whole	*.39*	.26	.22	.55	−.15	*.48*
Satisfaction with psychological health	.21	.22	.11	.28		.28
Anxiety	−.26	−.13	−.14	*−.41*	*−.36*	−.27
Depression	*−.33*	−.24	−.21	*−.44*		*−.38*
Bereavement stressors						
Short awareness of cancer						.10
Short awareness of impending death	.18			.15		.26
Poor quality of relationship with deceased parent	.28	.15	.20	.25	.21	.20
Poor quality of relationship with still living parent	.17		.19	.23		.13

Spearman’s correlation coefficients that correspond to the Cohen’s r effect size ≥ .1are presented; 0.1–0.3 small, 0.3–0.5 medium, and >0.5 large.

### Correlations Between Posttraumatic Growth and Bereavement Outcomes

Meaning in future life, satisfaction with life as a whole, satisfaction with psychological health, and lower levels of anxiety and depression were associated with all PTGI-domains except for Spiritual change. Overall, the associations were strongest in the domain’s Personal strength (r_s_ ≥ .28 or ≤ −.41) and Appreciation of life (r_s_ ≥ .28 or ≤ −.27). In these domain’s, a more positive outlook on future life and higher life satisfaction were associated with higher levels of growth while higher levels of anxiety and depressive symptoms were associated with lower levels of growth. Associations between bereavement outcomes and Spiritual change differed from the other associations in direction: believing more in a meaningful future life and having better life satisfaction were associated with lower levels of growth in this domain and satisfaction with psychological health and level of depression were not all associated with Spiritual change. The effect size range between small and medium but a large effect size was shown between satisfaction with life as a whole and Personal strength (r_s_ = .55) (Table 3).

### Correlations Between Posttraumatic Growth and Bereavement Stressors

Young adults who reported having had shorter awareness time of the cancer and impending death as well as a poorer quality in their relations to the dead parent also reported higher levels of posttraumatic growth in the different PTGI domains. The same results were found for those who experienced a poorer quality in their relations to their still living parent. However, the effect sizes were small (Table 3).

### Correlations Between Posttraumatic Growth and Sources Social Support

Those who reported having shared their grief with siblings and friends reported in general higher levels of posttraumatic growth. In contrast, those who had shared their grief with no one at all, or shared their grief with the parent before death, the still living parent, or partner, reported in general lower levels of posttraumatic growth in the different PTGI domains.

Further, higher number of sources of support and having received support from professionals were associated with higher levels of growth while no support was associated with lower levels of growth. Overall, the effect sizes were small, however a few medium effect sizes were shown between shared grief with partner and PTGI total (r_s_ = −.31), shared grief with partner and Personal strength (r_s_ = −.38), and shared grief with sibling and Appreciation of life (r_s_ = .30) (Table 4).

**Table 4.** Associations Between Posttraumatic Growth and Sources Of Social Support (*n* = 55).

	PTGI Tot	Relating to Others	New Possibilities	Personal Strength	Spiritual Change	Appreciation of Life
Shared grief with parent before death		−.14		−.16		−.11
Shared grief with still living parent	−.18		−.23	−.22		−.16
Shared grief with sibling	.20	.20				.30
Shared grief with partner	*−.31*	−.12	−.27	*−.38*	−.14	−.28
Shared grief with friends	.12	.25			.13	
Shared grief with no one	−.11	−.12				−.15
Received support from professional^a^	.14		.27	.25	.16	
Numbers of sources of support			.18	.13	.11	
Received no support	−.12	−.12				

Spearman’s correlation coefficients that correspond to the Cohen’s r effect size ≥ .1are presented; 0.1–0.3 small, 0.3–0.5 medium, and >0.5 large.

^a^A medical social worker or a psychologist.

## Discussion

The aim of the study was to examine posttraumatic growth and its associations with bereavement in a sample of young adults who lost a parent to cancer. Undoubtedly, this study shows that these young adults experienced posttraumatic growth, especially in the domains Personal strength and Appreciation of life. A shorter awareness time of the impending death and a poorer quality of relations with the parents were associated with more posttraumatic growth. Most associations with posttraumatic growth were found among the bereavement outcomes life satisfaction, a feeling of meaning in future life and psychological health. In fact, we argue that posttraumatic growth after struggling with a traumatic loss, could be described as an important bereavement outcome.

The present study shows, in line with previous research (Şimşek Arslan et al., 2022), that posttraumatic growth is possible after rumination also in this vulnerable group. The levels of posttraumatic growth in the present study correspond with the levels of posttraumatic growth in a Japanese study where young adults had lost their parent to cancer within the last 5 years (Hirooka, Fukahori, Akita, & Ozawa, 2017; Taku & McDiarmid, 2015). However, in the Japanese study the young adults had grown more in New possibilities and less in Personal strength compared with the present study (Hirooka, Fukahori, Ozawa, & Akita, 2017). This might be related to cultural differences that are of importance for behaviour after trauma. For example, Personal strength is measured by items related to self-reliance which is more typical of individualistic societies (Kashyap & Hussain, 2018) such as Sweden, while Japan more often is viewed as a collectivist society.

In the present study, no measure of posttraumatic stress symptoms was used, but anxiety and depression were negatively associated with posttraumatic growth which coincides with several studies (Meyerson et al, 2011). Indeed, the young adults who lost their parent in the present study experienced a traumatic event, which has also been described in previous studies by our research group (Lundberg et al., 2018a; 2018b; 2020; 2022). Other studies also supports the trauma of parental loss as it is associated with mortality and a long-lasting psychosocial health problems (Hiyoshi et al., 2021; Bylund-Grenklo et al., 2016). This trauma consists of multiple losses. Not only the parent’s unconditional love and care are lost, but also the person who cater security, provision, and support (Şimşek Arslan et al., 2022) leading to the loss of the assumptive world. In line with the theory of shattered assumptions (Janoff-Bulman, 1992) the young adults in the study had to reconstruct the way they look upon themselves and the world around them. A life they more or less taken for granted before is now more appreciated as well as they have discovered a personal strength they didn´t know they had before.

From a theoretical perspective, the prerequisite for posttraumatic growth is to experience trauma. Thus, psychosocial health problems should be expected at the same time as posttraumatic growth. Previous research has attempted to understand the associations between posttraumatic stress symptoms and posttraumatic growth and found evidence of an inverted u-curve relationship (Meyerson et al., 2011; Taku et al., 2015). Tentatively, the associations between anxiety and depression and posttraumatic growth could have similar relationship as between posttraumatic stress symptoms and posttraumatic growth. It is not surprising that positive bereavement outcomes, such as a positive outlook on life and life satisfaction, were positively related to posttraumatic growth in the present study. Norlander et al. (2007) has suggested that personality factors are of importance for posttraumatic growth among adults. et al. (2018a,b) has shown that the same group of young adults as in the present study had normal to high self-esteem; one of the intrapersonal factors of importance for bereavement outcomes (Stroebe et al., 2006). However, the study design does not allow for determining the causality in these relationships.

The present study demonstrates that also the source of support is of importance. Sharing grief with a sibling was related to growth, especially in Appreciation of life while sharing grief with a partner was related to less growth, especially in Personal strength. Provision of social support (Morris & Shakespeare-Finch, 2011) and especially the form of support has previously been found to be of importance for posttraumatic growth also among young adults (Meyerson et al., 2011; Zhou, Wu, & Zhen, 2018). According to the integrative risk factor framework for the prediction of bereavement outcome, interpersonal factors such as social resources, may promote resilience (Stroebe et al., 2006), which in turn contributes to the resistance of negative psychological outcomes (Rutter, 2007) of traumatic events. The comprehensive life changes taking place in the transition into adulthood also affects the relationship between siblings (Jewsbury Conger & Little, 2010). Sharing grief with a sibling who is probable to experience the same trauma as he/she him/herself also has lost the parent may increase the feeling of affinity with each other, as the grief is shared mutually, and this may increase the sense that life is valuable, thus leading to growth in appreciation of life. Receiving support from professionals was associated with more posttraumatic growth. The support of a professional may facilitate the coping process after parental death and acquiring professional support can provide opportunities for posttraumatic growth, also in line with earlier research (Şimşek Arslan et al., 2022). As argued by Şimşek Arslan et al. (2022), the professional support may contribute to the possibility to make meaning of the parent’s death.

Not only receiving support as the action of the bereaved person, but also taking part of supportive interactions is of importance for posttraumatic growth (Şimşek Arslan et al., 2022). All young adults in the present study took part in support groups before posttraumatic growth was measured. They were active participants, who in addition to receiving support also gave support and processed the loss. It could be suggested that the groups supported the young adults in their rumination which led to the ability to experience posttraumatic growth. In a metaanalysis, psychosocial interventions among individuals experiencing trauma have been suggested to increase posttraumatic growth. This, although the psychosocial interventions were not primarily targeting posttraumatic growth (Roepke, 2015).

## Methodological Considerations

As described in the integrative risk factor framework for the prediction of bereavement outcome (Stroebe et al., 2006), the bereavement process can be affected by a variety of factors that all interact with each other. The use of the framework has helped ensuring that relevant measures has been selected for the present study.

The homogeneous sample with most females who had lost a parent to cancer and who participated in a support group is somewhat problematic as it limits the generalizability to males. On the other hand, the findings should be generalised with carefulness as the sample size was small. Another problem with small sample studies is an increased risk of type 2 errors. For this reason, we have relied on Cohen’s effect size rather than statistical significance. This implies that no strong conclusions can be drawn about the population. Another factor that may impact the external validity is that the sample were recruited among young adults who had chosen to attend a support group which may have contributed to selection bias. Acceptance of support group attendance could be rejected both by those who feel most affected as well as those feeling less affected by the loss and experience of trauma is a prerequisite for post traumatic growth. Of these reasons, the results need therefore to be conformed in larger scaled studies before any strong conclusion can be drawn.

It could be assumed that the relatively short time, 1–1.5 years, may have affected the growth. Previous research is inconsistent whether time since death affects posttraumatic growth or not among young adults after parental loss. Some studies demonstrate that time is not a factor for posttraumatic growth (McClatchey, 2020; Salloum et al., 2017) while others have found that the longer time since death, the more growth (Brewer & Sparkes, 2011). Yet another study reports that the shorter the time since death the more growth (Wolshik et al., 2009).

Finally, the internal consistency reliability for the PTGI were acceptable for three of the domains. The internal consistency was low for the domains Spiritual change and Appreciation of life, a phenomenon also reported in other studies (Taku et al., 2015). The low internal consistency could be explained by them having the least number of items, two and three respectively (Streiner et al., 2015). However, it should be recognised that the sample size was small and that no strong conclusions can be drown about the Cronbach’s alpha in the present study.

## Clinical Implication

The results add important information about the young adults ability’ to experience posttraumatic growth after struggling with the loss of their parent. The study emphasizes the importance of support from professionals who indeed has the possibility to increase positive outcomes such as posttraumatic growth after parental loss. Thus, this vulnerable group needs to be recognised by the professionals, and it is important to also pay attention to and promote constructive rumination, thereby enhancing the development of positive psychological changes.

## Conclusion

This study shows that although parental loss certainly is a traumatic event, the struggle during the process of dealing with the loss may still lead to posttraumatic growth especially regarding appreciation of life and personal strength. From a theoretical perspective, these results may not be a surprise as the prerequisite for posttraumatic growth, to experience trauma, is well met when a parent dies, a trauma characterized by multiple losses. The results also add new information regarding the associations between posttraumatic growth and bereavement stressors, other bereavement outcomes and sources of social support. The result is of importance for professionals who supports these young adults in their clinical work.
